# Development and Validation of a New TaqMan Real-Time PCR for Detection of ‘*Candidatus* Phytoplasma pruni’

**DOI:** 10.3390/pathogens9080642

**Published:** 2020-08-07

**Authors:** Zala Kogej, Marina Dermastia, Nataša Mehle

**Affiliations:** Department of Biotechnology and Systems Biology, National Institute of Biology, Večna pot 111, 1000 Ljubljana, Slovenia; marina.dermastia@nib.si

**Keywords:** phytoplasma, X-disease, real-time PCR, *Prunus*

## Abstract

Phytoplasmas of the 16SrIII group are wide spread, and have a broad plant host range. Among these, ‘*Candidatus* phytoplasma pruni’ (‘*Ca.* P. pruni’; phytoplasmas of 16SrIII subgroup A) can cause serious diseases in *Prunus* species and ‘*Ca.* P. pruni’-related strains can infect other plant species, including grapevines. In this study, a new real-time PCR detection system was developed for ‘*Ca.* P. pruni’ using TaqMan chemistry. This test was designed to detect ‘*Ca.* P. pruni’, by amplifying the species-specific *secY* gene. In addition, a test to amplify the group-specific 16S rRNA gene region was also developed. The performances of both tests were evaluated. The test that amplifies the *secY* gene provided reliable and quick detection of ‘*Ca.* P. pruni’. Using the newly developed and validated test, ‘*Ca.* P. pruni’ was not found in any of the 434 field samples collected from different plants species grown in different regions of Slovenia.

## 1. Introduction

Phytoplasmas are wall-less plant pathogenic bacteria (class Mollicutes) that survive and multiply in the plant phloem and insect haemolymph. They are associated with diseases in several hundreds of plant species, which results in significant yield and quality losses in many economically important crops [1]. Phytoplasmas are categorised into 33 groups based on their highly conserved 16S ribosomal gene sequence [2]. The name ‘*Candidatus* (*Ca.*)’ represents the “unculturable” status of the phytoplasma and this status was approved in 1995 [3]. The name Phytoplasma was proposed in 1992 and adopted in 2004 [4]. The taxonomy of phytoplasma is complex and based on 16S ribosomal gene sequence as well as on biological, phytopathological, and genetic properties [4]. ‘*Ca*. Phytoplasma’ species can be differentiated when their 16S rRNA sequence have <97.5% similarity with other 16S rRNA gene sequences from previously described species [4].

The 16SrIII group phytoplasmas (i.e., the X-disease group) is wide spread and can damage a wide variety of economically important plants. The 16SrIII group has 26 subgroups (16SrIII-A to 16SrIII-Z) [2] and their hosts, vectors, symptoms, and geographic origin of the 16SrIII group phytoplasmas can differ greatly [5]. The most studied 16SrIII subgroup is 16SrIII-A. This subgroup includes ‘*Candidatus* Phytoplasma pruni’ (‘*Ca.* P. pruni’), which is associated with X-disease of stone fruit (*Prunus* species) [6,7,8]. ‘*Ca*. P. pruni’ has a unique DNA base composition, which classifies it as a ‘*Ca.* Phytoplasma’ species, and the major evolutionary divergence that divides it from the other 16SrIII subgroups is in the *secY* gene [9].

The economic importance of 16SrIII-A phytoplasmas is due to the complete loss of productivity and reduced life span of diseased *Prunus* trees [10]. Grafting of plants and comparison of DNA sequences from samples from eastern USA have shown that peach rosette, little peach, and red suture diseases are all closely related to Western X-disease [11]. Infections of *Prunus* species with ‘*Ca.* P. pruni’ have been found in eastern and western USA and eastern Canada [9]. 

In the 1950’s, X-disease was recognised as the most serious disease of peaches (*Prunus persica*) in western USA [12], and it was later shown to be transmitted by at least 15 species of leafhoppers [13]. In 1995, Van Steenwyk et al. [14] reported severe symptoms that almost eliminated cherry (*Prunus avium*) cultivation in some areas of California. X-disease can cause more than 50% mortality in *Prunus* species within three years after infection [15]. Other *Prunus* species that are susceptible to infection with ‘*Ca.* P. pruni’ include chokecherry (*Prunus virginiana*), sour cherry (*Prunus cerasus*), Japanese plum (*Prunus salicina*), almond (*Prunus dulcis*), apricot (*Prunus armeniaca*), nectarine (*Prunus persica* var. *nectarina*), Chinese bushcherry (*Prunus japonica*), Bessey cherry (*Prunus besseyi*), wild American plum (*Prunus americana*), wildgoose plum (*Prunus munsoniana*), and European plum (*Prunus domestica*) [12,16]. In the USA, ‘*Ca.* P. pruni’ has also been shown to infect apple trees (*Malus domestica*) [17]. 16SrIII-A phytoplasmas have also been reported in Napier grass (*Pennisetum purpureum*) in Ethiopia [18], and in cassava (*Manihot esculenta*) in Brazil [19] and Japan [20]. In northern Italy, 16SrIII-A phytoplasmas were found in two symptomatic cherry plants and in one out of nine analyzed specimens of *Philaenus spumarius* [21]. In addition, some ‘*Ca.* P. pruni’-related strains have been reported to cause damage to grapevines, including a variant of the 16SrIII-A subgroup (henceforth 16SrIII-A*) associated with North American grapevine yellows (NAGY) [22]. 

The diagnostics of phytoplasmas is difficult because they cannot be routinely grown in vitro. In addition, in woody plants their titres are low and vary according to season and plant organ [23,24]. Different approaches for their detection are now available, including biological tests, microscopy techniques, and immunological and molecular tests. Among these, the most reliable tests are PCR-based [25], e.g., amplification of 16S rRNA, *rp*, *secY*, *tuf,* or *groEL* genes [26]. Huang et al. [27] designed a real-time SYBR Green PCR test for detection of X-disease phytoplasmas in chokecherry. SYBR Green is fluorescent dye that binds non-specifically to double-stranded DNA, which can lead to false-positive results if the melting temperatures for each system are not compared to a reference strain. On the other hand, TaqMan chemistry can provide greater specificity by hybridisation of fluorogenic probes on the specific target regions, which we detect when it accumulates during PCR [25]. Therefore, the aim of the present study was to design a real-time PCR test based on TaqMan chemistry for the detection of ‘*Ca.* P. pruni’ in different hosts, which would be fast, easy, and reliable when screening high number of field samples. This test was then validated and used to screen different field samples from different parts of Slovenia.

## 2. Results

Real-time PCR tests were designed for the specific detection of ‘*Ca.* P. pruni’ and for the detection of other phytoplasmas from the 16SrIII group. For brevity and clarity, the ‘*Ca.* P. pruni’ test, the more specific test for detection of X-disease, is henceforth designated as the “sXd” test. The test to detect phytoplasmas from the whole 16SrIII group is a more general test as compared to the sXd test and is henceforth designated as the “gXd” test. The target genes were *secY* for the sXd test and the 16S rRNA gene for the gXd test. Both tests were also evaluated, and the sXd test was fully validated in accordance with PM 7/98 of the European and Mediterranean Plant Protection Organisation (EPPO) guidelines [28].

### 2.1. In Silico Analysis of the Designed Real-Time PCRs

**Figure 1:**
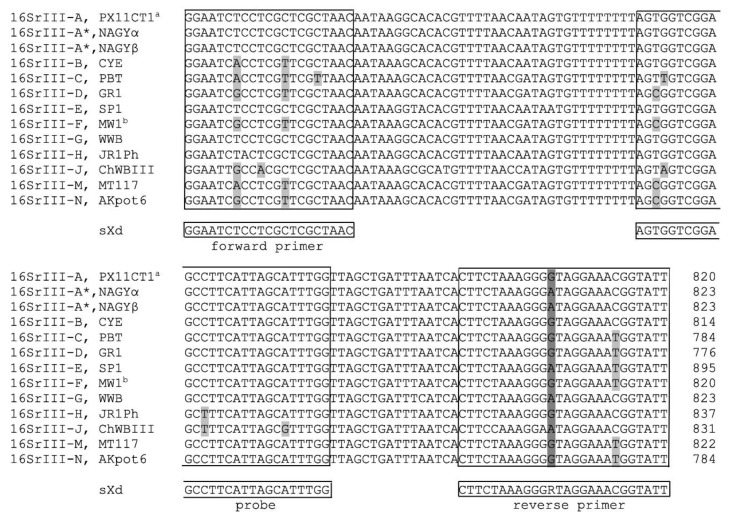
Design of the sXd test based on the *secY* gene. The alignment shows the *secY* gene for different representatives of the 16SrIII subgroups for which the sequences were available in the NCBI database. The sequences of the primers and probe are shown in the boxes below the alignment. The alignment sequences are from ‘*Ca*. P. pruni’ (PX11CT1; JQ268254), North American grapevine yellows sequevars α (NAGYα; KF853435) and β (NAGYβ; KF853451), Clover yellow edge phytoplasma (CYE; GU004332), Pecan bunchy top phytoplasma (PBT; GU004361), Goldenrod yellows phytoplasma (GR1; GU004364), Spirea stunt phytoplasma (SP1; GU004326), Milkweed yellows phytoplasma strain (MW1; GU004340), Walnut witches’ broom phytoplasma (WWB; JQ390054), Poinsettia branch-inducing phytoplasma (JR1Ph; GU004328), Chayote witches’ broom phytoplasma (ChWBIII; KP763468), Potato purple top phytoplasma (MT177; GU004333), and Potato purple top phytoplasma (AKpot6; GU004359). Light grey shading indicates differences in the sequences. Dark grey site indicates degenerative nucleotide R (G or A). ^a^ This sequence is the same for the 16SrIII-A strains CX-95 (JQ268249), WX-95 (JQ268250), PX92CT4 (JQ268251), PX92CT1 (JQ268252), and PX11CT2 (JQ268253); ^b^ This sequence is the same for the 16SrIII-F strains Vac (GU004360) and AKpot7 (GU004358).

The sequence alignment of the *secY* gene from the ‘*Ca.* P. pruni’ reference (PX11CT1; [9]) and representative strains of other 16SrIII subgroups was carried out to determine the specificity of the designed sXd test in silico (Figure 1). A degenerative nucleotide in the reverse primer was made, to cover variants of 16SrIII-A*, such as the NAGYα and NAGYβ sequevars. The Walnut witches’ broom phytoplasma (WWB) from 16SrIII-G has the same sequence as NAGYα and NAGYβ sequevars. When we implement the degenerative nucleotide, it was expected to be efficiently detected by the sXd test. At least one mismatch was seen in each subgroup when sequences from phytoplasmas belonging to other subgroups of 16SrIII were aligned (Figure 1). Additionally, the in silico specificity testing of the sXd amplicon with Basic Local Alignment Search Tool (BLAST) did not show non-specific hits for other phytoplasmas, bacteria, plant or insect hosts (data not shown).

**Figure 2:**
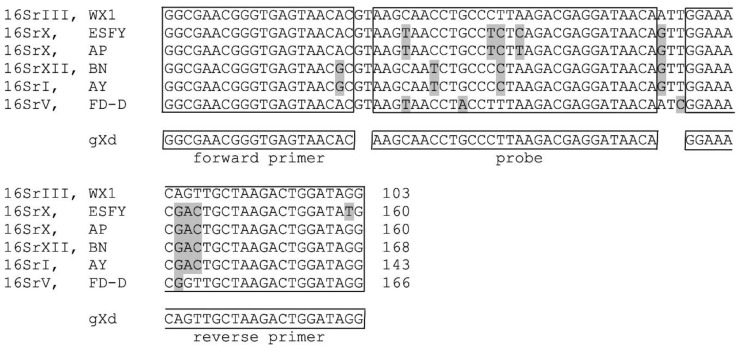
Design of the gXd test based on the 16S rRNA gene. The alignment shows part of the 16S rRNA gene of phytoplasmas from different 16Sr groups. The sequences of the primers and probe are shown in the boxes below the alignment. The alignment sequences are from Western X phytoplasma (WX1; FJ376628), *‘Ca*. P. prunorum’ (ESFY; AJ542544), ‘*Ca.* P. mali’ (AP; AJ542541), ‘*Ca.* P. solani’ (BN; AF248959), ‘*Ca.* P. asteris’ (AY; M30790) and Flavescence doree phytoplasma (FD-D; AY197644). Grey shading indicates differences in the sequences.

The primers and probe for the gXd test were designed based on the 16S rRNA gene of Western X phytoplasma (FJ376628). The region of the designed sequences of the primers and the probe are identical for the different 16SrIII-A isolates, and for the other 16SrIII phytoplasma subgroups, except for 16SrIII-J and 16SrIII-H. 16SrIII-J has a one-nucleotide difference in the probe region, and 16SrIII-H has a two-nucleotide difference in the reverse primer region (Supplementary Figure S1, Supplementary Table S1). The possibility to detect these two phytoplasma subgroups with the gXd test has not been experimentally tested, due to the lack of availability of the isolates. In silico analysis showed that phytoplasmas of other groups that are widespread in fruit trees and grapevines in Europe have at least three nucleotide mismatches with the designed primers and probe (Figure 2). BLAST search indicated potential cross-reactions with Sugarcane grassy shoot phytoplasma from 16SrXI, *‘Ca*. P. pini’ from 16SrXXI and ‘*Ca*. P. phoenicium’ from 16SrIX, due to the presence of just one nucleotide difference amplicon in the probe region of the gXd amplicon (Supplementary Figure S1, Supplementary Table S1).

### 2.2. Experimental Testing of the Specificities of the sXd and gXd Tests

The specificities of the sXd and gXd tests were evaluated through testing of (i) DNA from different phytoplasma isolates, (ii) DNA extracted from leaves of previously confirmed phytoplasma-free samples, and (iii) DNA extracted from leaves of naturally phytoplasma-infected grapevines and fruit trees sampled in different regions of Slovenia (Table 1). The sXd test was positive for the presence of 16SrIII-A phytoplasma in 100% of the 16SrIII-A isolates and the gXd test was positive for the presence of 16SrIII phytoplasmas in 100% of the 16SrIII isolates. Cross-reactivity with isolates belonging to subgroups 16SrIII-D, 16SrIII-E, and 16SrIII-F was observed with the sXd test. For these non-target isolates, the quantification cycles (Cq) obtained with the sXd test were higher (3–9 Cq) as compared to those obtained with the phytoplasma universal real-time PCR (maximum, 2 Cq). These differences in Cq are expected because of the differences in the nucleotide sequences between these non-target phytoplasmas and the sXd amplicon (Figure 1). No cross-reactivity was observed when using the sXd test on DNA from phytoplasmas belonging to other 16Sr groups and from any host plants (Table 1).

**Figure 3:**
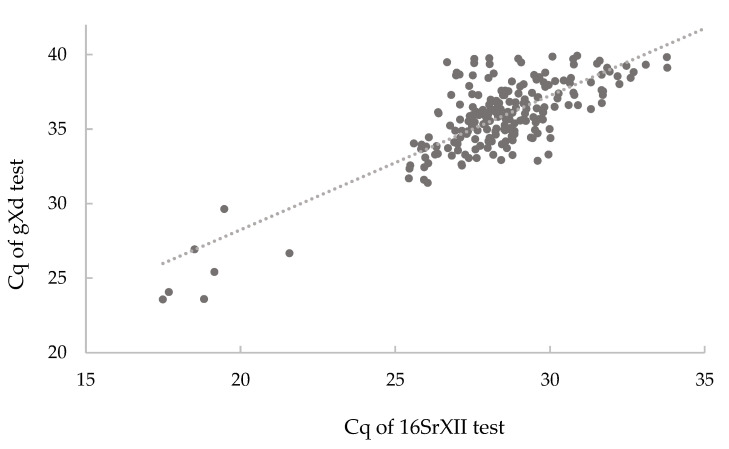
Correlation between Cq values obtained with the gXd test and the 16SrXII specific real-time PCR for samples infected with 16SrXII-A phytoplasmas.

With the gXd test, all of the isolates from the 16SrIII group were detected with the same efficiency, meaning that the Cq obtained with the gXd test was comparable to the Cq obtained with the universal phytoplasma real-time PCR (maximum difference, 1 Cq). Although not expected, the gXd test cross-reacted with many samples that were infected with the 16SrXII-A phytoplasmas. For all of these 16SrXII-A positive samples, the Cq values for the gXd test were higher by at least 7 Cq values, compared to the Cq values obtained for the universal phytoplasma real-time PCR (Table 1). In addition, similar differences were seen between the Cq values obtained with the 16SrXII specific real-time PCR and those obtained with the gXd test (Figure 3). No cross-reactivity was observed when using the gXd test on DNA from phytoplasmas belonging to other 16Sr groups and from any host plants (Table 1).

### 2.3. Analytical Sensitivity of the sXd Test

Three serial dilutions of 16SrIII-A phytoplasma DNA in the DNA extract of phytoplasma-free grapevine leaves were analyzed to determine the sensitivity of the sXd test (Table 2). Using the sXd test, the 16SrIII-A phytoplasmas were detected up to a dilution 10^7^. The sensitivity of the sXd test was comparable to the sensitivity of the universal phytoplasma real-time PCR (data not shown). The amplification efficiencies of the sXd test were between 97% and 99%.

### 2.4. Repeatability and Reproducibility of the sXd Test

The repeatability of the sXd test was evaluated by analyzing several replicates of DNA samples containing various concentrations of ‘*Ca*. P. pruni’. Three samples of 16SrIII-A infected plant materials (Green Valley X-disease—GVX, Peach yellow leaf roll—PYLR, and Western X—WX) were analyzed in the same run, with three replicates per dilution. The repeatability within a single dilution of each sample was high, with the standard deviation (SD) always below 1 Cq for the high, medium and low quantities of DNA (data not shown). 

**Figure 4:**
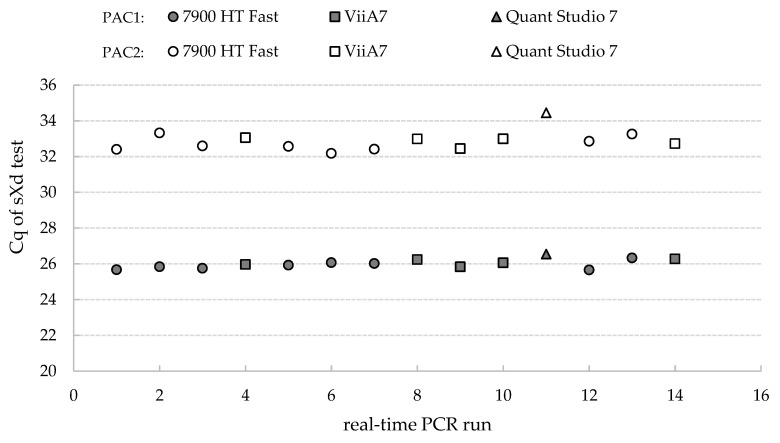
Run sequence plot of the repeated analysis for the sXd test of positive control samples with medium (PAC1) and low (PAC2) quantities of target DNA. The different real-time PCR machines are indicated: 7900 HT Fast (Applied Biosystems); ViiA7 (Applied Biosystems); and QuantStudio 7 (Applied Biosystems).

The reproducibility was analyzed for two dilutions of a 16SrIII-A positive DNA sample, with medium and low target concentrations. These were analyzed in different real-time PCR runs, with three different devices, and on 14 different days. The mean Cq values (±SD) of the samples with the medium and low target concentrations were 26.01 ± 0.26 and 32.87 ± 0.57, respectively (Figure 4).

## 3. Discussion

The majority of the methods used for detection of 16SrIII group are PCR based and restriction fragment length polymorphism analysis [5,9,29,30,31,32]. For more reliable detection of X-disease phytoplasmas in chokecherry, Huang et al. designed a real-time PCR based on SYBR Green chemistry [27]. In the present study, we propose the use of the sXd test to detect ‘*Ca*. P. pruni’ in different host plants, which is based on TaqMan chemistry. The sXd test was designed to target the *secY* gene, which is the most evolutionary divergent gene for distinguishing between different subgroups of the 16SrIII group [13]. Positive signals for the sXd test do not necessarily mean that a sample is infected with ‘*Ca*. P. pruni’, because the sXd test is not exclusively specific to 16SrIII-A phytoplasmas; thus, other subgroups of 16SrIII can also be detected. Therefore, for unambiguous confirmation of ‘*Ca*. P. pruni’ infection, additional identification tests are required for all samples that are positive with the sXd test. These tests can include direct and nested PCRs with the secYF1(III)/secYR1(III) primer pair, and later nucleotide sequence analysis [9].

The gXd test can also be included in the testing scheme, especially if other phytoplasmas of the 16SrIII group are of interest. However, caution must be taken with interpretation of the results, because the gXd test cross-reacts with 16SrXII-A, and probably also with Sugarcane grassy shoot phytoplasma, ‘*Ca*. P. pini’ and ‘*Ca*. P. phoenicium’. Cross-reactivity with 16SrXII-A positive samples was unexpected, because the in silico analyses showed six nucleotide differences between the gXd amplicon and the correlating part of the 16S rRNA gene of ‘*Ca*. P. solani’, in both the primers and probe regions. The same sequence in this region as the reference strain of ‘*Ca.* P. solani’ (AF248959) has also ‘*Ca.* P. asteris’ reference strain (M30790). However, positive signals were obtained when testing ‘*Ca.* P. solani’ strains with the gXd test, but not when testing the Aster yellows (AY) group positive samples. The AY group strains share 97% to 100% 16S rRNA sequence similarity [33], and some of the strains (especially from 16SrI-A) have one to three more mismatches with the gXd amplicon as compared to the reference ‘*Ca.* P. asteris’. Most of ‘*Ca.* P. solani’ 16S rRNA sequences from different strains [34] do not cover this part of sequence, so it is not possible to tell if some strains might have fewer mismatches with the gXd amplicon, which might be what gives the positive signals.

The differences between the Cq values of the sXd and gXd tests and the universal phytoplasma real-time PCR can be used to estimate the probability of infection with ‘*Ca*. P. pruni’ or with phytoplasmas of other groups or subgroups. However, this is not a reliable diagnostics parameter, because plants can be simultaneously infected with phytoplasmas from different 16Sr groups [35,36,37,38]. So, for the present study, we can unambiguously conclude that none of the field samples contained ‘*Ca.* P. pruni’. Nevertheless, for the above-mentioned reasons, there is a possibility that some samples were infected with not just 16SrXII phytoplasmas, but also phytoplasmas from other 16SrIII subgroups. This could be examined through sequencing of cloned PCR products, obtained from the phytoplasma universal PCR. 

Early and accurate detection of ‘*Ca.* P. pruni’ is needed to prevent the spread of their related diseases. Here, the developed and validated sXd test is shown to be a reliable and sensitive test that is useful for fast screening for ‘*Ca*. P. pruni’ in large numbers of field samples.

## 4. Materials and Methods

### 4.1. Plant Aaterials

In this study, DNA samples of phytoplasmas from different groups and subgroups were included. They were kindly provided by Dr X. Foissac (French National Institute for Agriculture, Food, and Environment (INRA), Villenave d’Ornon, France), Dr A. Bertaccini (University of Bologna, Bologna, Italy), Dr G. Brader (Austrian Institute of Technology (AIT), Vienna, Austria) and Dr J. Jović (Institut za zaštitu bilja i životnu sredinu (IZBIS), Beograd, Serbia) (Table 1).

Leaves showing symptoms of phytoplasma diseases were sampled from 422 grapevines, 5 peach trees, 2 apricot trees, 3 apple trees, 1 pear tree, and 1 plum tree, and were collected between 2010 and 2019 in different locations in Slovenia. They were collected in the framework of official monitoring for the grapevine yellows and apple proliferation (16SrX) groups of phytoplasmas, which was led by the Administration of the Republic of Slovenia for Food Safety, Veterinary Sector, and Plant Protection. The grapevine samples were collected from 24 different cultivars grown in the main wine growing regions of Slovenia. Samples were collected in at least three different places from plants exhibiting typical symptoms of phytoplasma infection. Mid-vein tissue from different leaves from tree/grapevine were cut out and stored at −20 °C.

### 4.2. DNA Extraction and Detection of Phytoplasmas

Total DNA was isolated from 1 g leaf mid-vein tissue, which was homogenised with a FastPrep instrument (MP Biomedicals, Illkirch-Graffenstaden, France) using kits (QuickPick Plant DNA kits; Bio-Nobile, Pargas, Finland) and a purification system (KingFisher mL; Thermo Scientific, Waltham, MA, USA) [39]. The total DNA extracted from each sample was used as the template for universal phytoplasma real-time PCR to amplify the phytoplasma 16S rRNA gene [40]. The final 10-μL reaction volumes for the real-time PCR contained 2 μL 10-fold diluted DNA sample, TaqMan Universal Master Mix (Applied Biosystems, Foster City, CA, USA), 300 nM forward primer, 900 nM reverse primer, and 100 nM probe. The real-time PCR was carried out in 348-well plates (Applied Biosystems) using three different detection systems: a 7900HT Fast Real-Time PCR system (Applied Biosystems); a ViiA 7 system (Applied Biosystems); and a QuantStudio 7 system (Applied Biosystems). The cycling conditions were 2 min at 50 °C, 10 min at 95 °C, 45 cycles of 15 s at 95 °C, and 1 min at 60 °C. Samples were considered positive if the target DNA was detected in at least two of three replicates, with Cq values < 40, and if the negative isolation and amplification controls were negative. Depending on the real-time PCR machine, software SDS 2.4 and QuantStudio Real-Time PCR software 1.3 (both Applied Biosystems) were used for fluorescence acquisition and calculation of Cq. The baseline was always set automatically, and the threshold manually, as 0.065. The identification of the phytoplasmas other than 16SrIII group was carried out using real-time PCR according to protocols for specific detection of: the “stolbur” (16SrXII) group, which includes phytoplasmas that cause Bois noir [41,42], AY (16SrI) group [43] and apple proliferation (16SrX) [44,45]. The 18S rRNA primer–probe mix (Applied Biosystems) was used as the control, to evaluate the quality of the DNA in the DNA extraction [39].

### 4.3. Design and Set-up of the sXd and gXd Tests

The *secY* gene sequences of ‘*Ca.* P. pruni’ and phytoplasmas from other 16SrIII subgroups available in the NCBI database (Figure 1) were aligned using the Vector NTI Suite 9 software (Thermo Scientific, Waltham, MA, USA), to find suitable regions for primer and probe design. The search for optimal sets of primers and the probe was carried out using Primer Express (Applied Biosystems). Secondary structure formation in the designed amplicon was tested using the OlygoAnalyzer tool (Integrated DNA Technologies, Inc., Coralville, Iowa, USA), and the specificity of binding with BLAST searches of public databases. In the text, the test is referred to as the sXd test, which indicates the use of primer pair sXd-F (5′-GGAATCTCCTCGCTCGCTAAC-3′) and sXd-R (5′-AATACCGTTTCCTAYCCCTTTAGAAG-3′), and the use of probe sXd-P (5′FAM-AGTGGTCGGAGCCTTCATTAGCATTTGG-3′NFQ).

Additionally, the gXd test that was designed to detect the whole 16SrIII group, was based on the 16S rRNA gene using 16S rRNA gene sequences. The 16SrIII-A sequences were compared with 16S rRNA sequences from the other 16Sr groups (Figure 2) and with other subgroups of 16SrIII (Supplementary Figure S1, Supplementary Table S1). This test included primer pair gXd-F (5′-GGCGAACGGGTGAGTAACAC-3′) and gXd-R (5′-CCTATCCAGTCTTAGCAACTGTTTCC-3′), and the probe gXd-P (5′FAM-AAGCAACCTGCCCTTAAGACGAGGATAACA-3′NFQ), and is hence referred to as the gXd test.

The designed primers and probes were used in real-time PCRs. The reactions were performed in 10-μL final volumes that contained 2 μL 10-fold diluted DNA sample, TaqMan Universal Master Mix (Applied Biosystems), 900 nM forward and reverse primers, and 250 mM probe. These PCR reactions were run as described above. 

### 4.4. Validation of the sXd and gXd Tests

The specificities of the sXd and gXd amplicons were tested both in silico and experimentally, using the DNA of different phytoplasmas and the DNA of different phytoplasma-free host plants (Table 1). Further full validation according to the EPPO recommendations [28] was only carried out for the sXd test.

The sensitivity of the sXd test was determined on three samples of DNA extracts of phytoplasmas belonging to 16SrIII-A (i.e., GVX, PYLR, and WX). The samples were diluted up to 10^9^-fold in DNA of phytoplasma-free grapevines. Each dilution was tested as three replicates. The dynamic range was determined, and in this range, the slope was calculated for the linear regression line (k) between the logarithmic values of the relative DNA concentrations and the Cq values. This slope was used to determine the amplification efficiency (E = (10^[−1/k]^) − 1), where a value of 1.0 indicates 100% amplification efficiency [46]. The squared regression coefficient was also determined (R^2^).

The repeatability of the sXd test was evaluated through analysis of three replicates of DNA samples with low, medium, and high concentrations of the target. To determine the reproducibility of the sXd test, one sample with medium and one sample with low concentrations of ‘*Ca.* P. pruni’ were analyzed. Both of these samples were tested on 14 different days and with three different real-time PCR machines (i.e., ABI 7900 HT Fast, ViiA7, Quant Studio 7). Different runs were performed with freshly prepared reaction mix. Between the runs, at least one condition was changed, in terms of different real-time PCR machine, different lots of chemicals, and different day.

## Figures and Tables

**Table 1:** Results of the analysis of the target and non-target phytoplasmas with the different real-time PCR tests.

Host Plant/Insect	Phytoplasma	Disease	16Sr Group	DNA Source	No. of Samples *	Universal Phytoplasma Real-Time PCR (Cq)	sXd Test (Cq)	gXd Test (Cq)
*Catharanthus roseus*	*‘Ca.* P. pruni’	Peach Western X	III-A	DNA, Foissac	1	17	18	17
Unknown		Green Valley X-disease	III-A	DNA, Bertaccini	1	18	20	18
Unknown		Peach yellow leaf roll	III-A	DNA, Bertaccini	1	19	20	19
Unknown		Western X	III-A	DNA, Bertaccini	1	18	20	19
*Solidago rugosa*		Golden rod yellows	III-D	DNA, Foissac	1	19	28	19
*Spiraea tomentosa*		Spirea stunt	III-E	DNA, Foissac	1	17	20	17
*Asclepias syriaca*		Milkweed yellows	III-F	DNA, Foissac	1	18	26	18
Mix of *Vitis vinifera, Hyalesthes obsoletus,* *Catharanthus roseus, Urtica dioica*	‘*Ca*. P. solani’	Bois noir	XII-A	DNA, Brader	1	16	Negative	24
Mix of *Hyalesthes obsoletus,* *Catharanthus roseus*					1	16	Negative	24
Mix of *Hyalesthes obsoletus, Catharanthus roseus, Agallia ribauti*					1	17	Negative	27
Mix of *Vitis vinifera, Catharanthus roseus, Agallia ribauti, Reptalus panzeri*					1	18	Negative	30
*Vitis vinifera*					1	26	Negative	33
*Zea mays*	*‘Ca*. P. solani’	Maize redness	XII-A	DNA, Jović	1	17	Negative	25
					1	17	Negative	24
*Reptalus panzeri*					1	24	Negative	31
*Catharanthus roseus*	*‘Ca*. P. solani’		XII-A	DNA, Foissac	1	20	Negative	27
	‘*Ca*. P. ulmi’		V-A	DNA, Foissac	1	22	Negative	Negative
	‘*Ca*. P. rubi’		V-E	DNA, Foissac	1	20	Negative	Negative
	Aster yellows group		I	Plant, tissue culture	2	20–27	Negative	Negative
*Vitis vinifera*	*‘Ca*. P. solani’	Bois noir	XII-A	Plant, field sampling (2018)	10	25–27	Negative	38–40
					45	26–32	Negative	Negative
				Plant, field sampling (2019)	188	24–32	Negative	32–40
					30	25–34	Negative	Negative
*Vitis vinifera*		Flavescence dorée and Bois noir	V and XII-A	Plant, field sampling (2018)	10	23–30	Negative	Negative
				Plant, field sampling (2019)	4	25–28	Negative	34–39
					19	23–30	Negative	Negative
*Vitis vinifera*		Flavescence dorée	V	Plant, field sampling (2015)	1	25	Negative	Negative
				Plant, field sampling (2018)	23	24–33	Negative	Negative
				Plant, field sampling (2019)	90	21–31	Negative	Negative
*Vitis vinifera*	Aster yellows group		I	Plant, field sampling (2019)	1	28	Negative	Negative
*Prunus persica*	‘*Ca*. P. prunorum’	European stone fruit yellows	X-B	Plant, field sampling (2010)	1	26	Negative	Negative
				Plant, field sampling (2018)	2	24–28	Negative	Negative
				Plant, field sampling (2019)	1	24	Negative	Negative
*Prunus armeniaca*	*‘Ca*. P. prunorum’	European stone fruit yellows	X-B	Plant, field sampling (2018)	1	24	Negative	Negative
				Plant, field sampling (2019)	1	27	Negative	Negative
*Malus domestica*	‘*Ca*. P. mali’	Apple proliferation	X-A	Plant, field sampling (2010)	2	28–32	Negative	Negative
*Pyrus communis*	‘*Ca*. P. pyri’	Pear decline	X-C	Plant, field sampling (2018)	1	24	Negative	Negative
*Prunus persica*	Negative for phytoplasmas			Plant, field sampling (2018)	1	Negative	Negative	Negative
*Prunus* subg. *Prunus*				Plant, field sampling (2016)	1	Negative	Negative	Negative
*Malus domestica*				Plant, field sampling (2018)	1	Negative	Negative	Negative
*Vitis vinifera*				Plant, field sampling (2018)	1	Negative	Negative	Negative

* Where there was just one sample, the Cq value is the mean of three replicates; if there were more samples, the Cq range is given (min–max).

**Table 2:** Efficiency of the sXd test.

16SrIII-A Isolate	Dilution Factors		Linear Regression		
	Range of Detection	Dynamic Range	k	R^2^	E
Green Valley X-disease	10^−^^1^–10^−^^7^	10^−^^1^–10^−^^6^	−3.389	0.998	0.973
Peach yellow leaf roll	10^−^^1^–10^−^^7^	10^−^^1^–10^−^^5^	−3.386	0.999	0.974
Western X	10^−^^1^–10^−^^7^	10^−^^1^–10^−^^6^	−3.358	0.998	0.985

k, slope of the linear regression line in the plot of Cq against log [relative concentration]; R^2^, mean square regression coefficient; and E, efficiency of amplification.

## Supplementary Materials

The following are available online at https://www.mdpi.com/2076-0817/9/8/642/s1, Figure S1: Alignment to gXd test and phytoplasmas from the different groups and the 16SrIII subgroups. Table S1: GenBank numbers of the aligned sequences indicated in Supplementary Figure S1.
