# Supplementary material for: Development and Validation of a New TaqMan Real-Time PCR for Detection of ‘Candidatus Phytoplasma pruni’
Source: Pathogens. 2020 Aug 7;9(8):642. doi: 10.3390/pathogens9080642 (PMC7459698; doi:10.3390/pathogens9080642)
Supplement: Supplementary file 1 [file pathogens-09-00642-s001.pdf]

Supplementary Materials

```
16SrIII-A   GGCGAACGGGTGAGTAACACGTAAGCAACCTGCCCTTAAGACGAGGATAACAATTGGAAA
16SrIII-B   GGCGAACGGGTGAGTAACACGTAAGCAACCTGCCCTTAAGACGAGGATAACAATTGGAAA
16SrIII-C   GGCGAACGGGTGAGTAACACGTAAGCAACCTGCCCTTAAGACGAGGATAACAATTGGAAA
16SrIII-D   GGCGAACGGGTGAGTAACACGTAAGCAACCTGCCCTTAAGACGAGGATAACAATTGGAAA
16SrIII-E   GGCGAACGGGTGAGTAACACGTAAGCAACCTGCCCTTAAGACGAGGATAACAATTGGAAA
16SrIII-F   GGCGAACGGGTGAGTAACACGTAAGCAACCTGCCCTTAAGACGAGGATAACAATTGGAAA
16SrIII-G   GGCGAACGGGTGAGTAACACGTAAGCAACCTGCCCTTAAGACGAGGATAACAATTGGAAA
16SrIII-H   GGCGAACGGGTGAGTAACACGTAAGCAACCTGCCCTTAAGACGAGGATAACAATTGGAAA
16SrIII-J   GGCGAACGGGTGAGTAACACGTAAGCAACCTACCCTTAAGACGAGGATAACAATTGGAAA
16SrIII-L   GGCGAACGGGTGAGTAACACGTAAGCAACCTGCCCTTAAGACGAGGATAACAATTGGAAA
16SrIII-M   GGCGAACGGGTGAGTAACACGTAAGCAACCTGCCCTTAAGACGAGGATAACAATTGGAAA
16SrIII-N   GGCGAACGGGTGAGTAACACGTAAGCAACCTGCCCTTAAGACGAGGATAACAATTGGAAA
16SrIII-O   GGCGAACGGGTGAGTAACACGTAAGCAACCTGCCCTTAAGACGAGGATAACAATCGGAAA
16SrIII-P   GGCGAACGGGTGAGTAACACGTAAGCAACCTGCCCTTAAGACGAGGATAACAATTGGAAA
16SrIII-Q   GGCGAACGGGTGAGTAACACGTAAGCAACCTGCCCTTAAGACGAGGATAACAATTGGAAA
16SrIII-S   GGCGAACGGGTGAGTAACACGTAAGCAACCTGCCCTTAAGACGAGGATAACAATTGGAAA
16SrIII-V   GGCGAACGGGTGAGTAACACGTAAGCAACCTGCCCTTAAGACGAGGATAACAATTGGAAA
16SrIII-W   GGCGAACGGGTGAGTAACACGTAAGCAACCTGCCCTTAAGACGAGGATAACAATTGGAAA
16SrIII-X   GGCGAACGGGTGAGTAACACGTAAGCAACCTGCCCTTAAGACGAGGATAACAATTGGAAA
16SrIII-Y   GGCGAACGGGTGAGTAACACGTAAGCAACCTGCCCTTAAGACGAGGATAACAATTGGAAA
16SrXXI     GGCGAACGGGTGAGTAACACGTAAGCAACCTGCCTTTAAGACGAGGATAACAATTGGAAA
16SrIX      GGCGAACGGGTGAGTAACACGTAAGCAACCTGCCTTTAAGACGAGGATAACAATTGGAAA
16SrXI      GGCGAACGGGTGAGTAACACGTAAGCAACCTGCCTTTAAGACGAGGATAACAATTGGAAA

gXd         GGCGAACGGGTGAGTAACAC  AAGCAACCTGCCCTTAAGACGAGGATAACA    GGAAA
               forward primer                   probe

16SrIII-A   CAGTTGCTAAGACTGGATAGG  121
16SrIII-B   CAGTTGCTAAGACTGGATAGG  162
16SrIII-C   CAGTTGCTAAGACTGGATAGG  103
16SrIII-D   CAGTTGCTAAGACTGGATAGG  102
16SrIII-E   CAGTTGCTAAGACTGGATAGG  162
16SrIII-F   CAGTTGCTAAGACTGGATAGG  162
16SrIII-G   CAGTTGCTAAGACTGGATAGG  162
16SrIII-H   CAATCGCTAAGACTGGATAGG  162
16SrIII-J   CAGTTGCTAAGACTGGATAGG  162
16SrIII-L   CAGTTGCTAAGACTGGATAGG  115
16SrIII-M   CAGTTGCTAAGACTGGATAGG  137
16SrIII-N   CAGTTGCTAAGACTGGATAGG  137
16SrIII-O   CAGTTGCTAAGACTGGATAGG  137
16SrIII-P   CAGTTGCTAAGACTGGATAGG  137
16SrIII-Q   CAGTTGCTAAGACTGGATAGG  162
16SrIII-S   CAGTTGCTAAGACTGGATAGG  167
16SrIII-V   CAGTTGCTAAGACTGGATAGG  162
16SrIII-W   CAGTTGCTAAGACTGGATAGG  162
16SrIII-X   CAGTTGCTAAGACTGGATAGG  162
16SrIII-Y   CAGTTGCTAAGACTGGATAGG  137
16SrXXI     CAGTTGCTAAGACTGGATAGG  164
16SrIX      CAGTTGCTAAGACTGGATAGG  137
16SrXI      CAGTTGCTAAGACTGGATAGG  130

gXd         CAGTTGCTAAGACTGGATAGG
              reverse primer
```

Figure S1. Alignment of sequences of phytoplasmas belonging to the 16SrIII subgroups and different other groups for the gXd test design. The sequences of the primers and probes are shown in the boxes below the alignment. Grey shading indicates differences between the nucleotide sequences the primer and probe regions. The GenBank numbers of the aligned sequences are shown in the Supplementary Materials Table S1. Where there is more than one strain per subgroup in the Table S1, the sequence of the studied part of the 16S rRNA gene was the same for all strains of that subgroup.

Table S1. GenBank numbers of the aligned sequences indicated in Supplementary Figure S1.

16Sr group	Phytoplasma	Strain	GenBank N°
16SrIII-A	'*Ca*. P. pruni'	PX11CT1	JQ044393, JQ044392
		PX11CT2	JQ044391, JQ044390
		PX92CT1	JQ044389, JQ044388
		PX92CT2	JQ044387, JQ044386
		PX92CT3	JQ044385, JQ044384
		PX92CT4	JQ044383, JQ044382
		CX-95	JQ044397
		WX-95	JQ044396
	Western X phytoplasma strain	WX1	FJ376628
	Canadian peach X mycoplasma-like organism	CX	L33733
16SrIII-B	Clover yellow edge phytoplasma	CYE	AF175304
16SrIII-C	Pecan bunch phytoplasma strain	PB1	FJ376626
16SrIII-D	Goldenrod yellows phytoplasma	GRY-GR1	FJ376627
16SrIII-E	Spiraea stunt phytoplasma	SP1	AF190228
16SrIII-F	Milkweed yellows phytoplasma	MW1	AF510724
	Vaccinium witches′ broom	VAC	X76430
	Potato purple top phytoplasma	AKpot7	GU004370
6SrIII-G	Walnut witches' broom phytoplasma	WWB	JQ044395, JQ044394
16SrIII-H	Poinsettia branch-inducing phytoplasma	PoiBI	AF190223
16SrIII-J	Chayote witches' broom phytoplasma	ChWBIII(Ch10)	AF147706
16SrIII-L	Cassava frogskin disease phytoplasma	FSDY15	EU346761
16SrIII-M	Montana potato purple top phytoplasma	PPT-MT117-1	FJ226074
16SrIII-N	Alaska potato purple top phytoplasma	PPT-AK6	GU004365
16SrIII-P	Dandelion virescence phytoplasma	DanVir	AF370119
16SrIII-Q	Black raspberry witches'-broom phytoplasma	BRWB7	AF302841
16SrIII-S	Western peach X-disease	WX	L04682
16SrIII-V	Passion fruit witches'-broom phytoplasma	PassWB-Br4	GU292082
16SrIII-W	Heterothalamus little leaf	HetLL	KC412029
16SrIII-X	*′Conyza bonariensis′* phytoplasma	ConWBA	KC412026
16SrIII-Y	Cranberry false blossom	CB1A	KF626525
16SrXXI	'*Ca*. P. pini'	P127	AJ632155
16SrIX	'*Ca*. P. phoenicium'	A4	AF515636
16SrXI	Sugarcane grassy shoot phytoplasma	MZN5	KP746933
